# A Comparison of Immersive Realities and Interaction Methods: Cultural Learning in Virtual Heritage

**DOI:** 10.3389/frobt.2019.00091

**Published:** 2019-09-24

**Authors:** Mafkereseb Kassahun Bekele, Erik Champion

**Affiliations:** UNESCO Chair of Cultural Heritage and Visualisation, School of Media, Creative Arts and Social Inquiry, Curtin University, Bentley, WA, Australia

**Keywords:** mixed reality, collaborative interaction, multimodal interaction, virtual heritage, cultural learning

## Abstract

In recent years, Augmented Reality (AR), Virtual Reality (VR), Augmented Virtuality (AV), and Mixed Reality (MxR) have become popular immersive reality technologies for cultural knowledge dissemination in Virtual Heritage (VH). These technologies have been utilized for enriching museums with a personalized visiting experience and digital content tailored to the historical and cultural context of the museums and heritage sites. Various interaction methods, such as sensor-based, device-based, tangible, collaborative, multimodal, and hybrid interaction methods, have also been employed by these immersive reality technologies to enable interaction with the virtual environments. However, the utilization of these technologies and interaction methods isn't often supported by a guideline that can assist Cultural Heritage Professionals (CHP) to predetermine their relevance to attain the intended objectives of the VH applications. In this regard, our paper attempts to compare the existing immersive reality technologies and interaction methods against their potential to enhance cultural learning in VH applications. To objectify the comparison, three factors have been borrowed from existing scholarly arguments in the Cultural Heritage (CH) domain. These factors are the technology's or the interaction method's potential and/or demonstrated capability to: (1) establish a contextual relationship between users, virtual content, and cultural context, (2) allow collaboration between users, and (3) enable engagement with the cultural context in the virtual environments and the virtual environment itself. Following the comparison, we have also proposed a specific integration of collaborative and multimodal interaction methods into a Mixed Reality (MxR) scenario that can be applied to VH applications that aim at enhancing cultural learning *in situ*.

## Introduction

The benefits of immersive reality technologies and Human-Computer-Interaction (HCI) methods for the preservation, representation and dissemination of cultural heritage have been widely researched in CH (Addison and Gaiani, 2000; Papagiannakis et al., 2008; Adhani and Rambli, 2012; Anthes et al., 2016; Bekele et al., 2018). Although critical technical limitations, such as lack of robust and real-time tracking and lack of intuitive interaction interfaces, hinder users' experience, immersive reality technologies have achieved a fascinating acceptance in various application areas of VH (Carrozzino and Bergamasco, 2010). This trend has resulted in an increasing utilization of immersive reality and HCI methods in the contemporary museums, tourism industry, and the VH domain. The dissemination of these technologies within traditional museums and heritage sites, however, has been challenged by a number of factors, such as its cost of installation, and demand of high-end computers and programming expertise (Carrozzino and Bergamasco, 2010). Furthermore, the technology keeps advancing quite often, meaning cultural institutions, and professional need to acquire the new technologies and the appropriate skills for content development. In the last few years, however, a significant number of affordable immersive reality headsets and hand-held devices equipped with a higher graphical computation, positional tracking sensors, and rendering capability are changing the trend. As a result, immersive reality technologies and HCI methods are being exploited for educational, explorative, and exhibition enhancement purposes (Scott et al., 2018; Zhao et al., 2018). Eventually, such developments can change the position of traditional museums and heritage sites toward accommodating the installation of immersive reality technologies. However, an effective utilization of these technologies needs to be supported by informed practical guidelines. In this regard, this paper will present a comparison of AR, VR, AV, and MxR technologies and HCI methods that are commonly adopted in VH applications. A similar comparison of immersive environments has been attempted by Kateros et al. (2015). However, the authors focused on gamified VR and HCI rather than the full spectrum of the reality-virtuality continuum. Our paper, on the other hand, attempts to compare the whole spectrum and a wider range or interaction methods in order to assist in predetermining their relevance to VH applications. In addition, the paper attempts to identify the best approach in terms of integrating a specific form of immersive reality and interaction method to enable cultural learning in a specific VH scenario.

The remainder of this paper is organized as follows. Section Immersive reality technologies discusses the segments of the reality-virtuality continuum and their enabling technologies. Different categories of interaction methods are discussed as an aspect of immersive reality enabling technologies under this section. Section Comparing Immersive Realities and Interaction Interfaces provides a comparison of immersive realities and interaction interfaces against three factors (contextual relationship, collaboration, and engagement) borrowed from existing scholarly arguments in the CH domain. Following the comparison, the section will also provide suggestions as to which forms of immersive reality and interaction methods can enhance cultural learning in VH applications. Finally, section Conclusion provides a conclusion and summarizes the paper.

## Immersive Reality Technologies

In the past, immersivity and presence have been associated with or regarded as indicators of a successful VR application due to the technological constraints that made immersivity a unique quality of VR. As a result, the applicability of such aspects hasn't been realized in AR and MxR applications until recently. However, the recent advances in Head-Mounted-Displays (HMDs) enable audio-visual immersivity in all of the segments of the reality-virtuality continuum. For instance, one of the recent HMDs “Microsoft HoloLens,” which is built mainly for an AR/MxR experience, can also be used for VR scenarios. Such potentials are changing the trend of the enabling technologies behind immersive reality in terms of establishing a versatile platform where any segment of the continuum can be implemented upon. Hence, it is crucial to discuss immersive reality from two different perspectives: (1) focusing on its forms (categories), and (2) focusing on its enabling technologies.

### Forms of Immersive Reality

Augmented Reality (AR), Virtual Reality (VR), Augmented Virtuality (AV), and Mixed Reality (MxR) are specific segments of the reality-virtuality continuum. In order to avoid a repetitive appearance of “AR/VR/AV/MxR,” the term “immersive reality” will serve as a collective term representing these segments. However, when there is an explicit reference to a specific segment, the appropriate term will be used.

Azuma (1997) defined AR as “a system that combines real and virtual content, provides a real-time interactive environment, and registers in 3D.” In general, AR aims to enhance our understanding or perception of the physical environment. This could be achieved by adding digital content to our view of the physical environment or by virtually erasing some parts of our view. The adoption of AR into VH began in early 2000s. The ARCHEOGUIDE project is a typical example (Vlahakis et al., 2001). Over the las decade, following the availability of relatively affordable immersive reality devices studies in the VH domain have established AR as a system that enhances users' view and understanding of CH assets (Liarokapis et al., 2005; Kim et al., 2009; Zoellner et al., 2009; Haydar et al., 2011; Damala and Stojanovic, 2012; Casella and Coelho, 2013; Rattanarungrot et al., 2014; D'Auria et al., 2015; Leach et al., 2018).

Virtual Reality (VR), on the other hand, transports users to a highly immersive virtual environment without any or little possibility of directly interacting with their immediate physical surroundings (Carmigniani et al., 2011). VR has the potential to simulate imaginative and existing physical environments along with their processes. The simulations can be tuned to a highest level of multisensorial realism in order to affect users' visual, auditory, tactile, vestibular, and even olfactory and gustatory senses (Zhao, 2009). VH applications have extensively employed VR for virtual reconstruction, simulation, educational, and explorative themes (Gaitatzes et al., 2001; Mourkoussis et al., 2002; Christou et al., 2006; Haydar et al., 2011; Pietroni et al., 2013).

Similar to AR, Augmented Virtuality (AV) also attempts to enhance users' understanding of the environment it is applied to. To this effect, AV augments virtual environments with live scenes of events and elements from the real-world. Due to virtual simulations serving as the base environment in AV, this segment could be misunderstood as a variation of VR. This is problematic since the whole purpose of augmenting virtual environments with live scenes is to enhance our understanding of the underlying virtual environment, which diverts from VR's aim. Furthermore, VR has no direct implication on our perception of the real world, which to some extent AV achieves since live scenes are streamed from the real world. Interaction and presence in a virtual environment that simulates the physical world in real time might indirectly influence our perception of the physical reality. AV applications are very rare due to the technical challenge of tracking the pose of elements from the real-world and the difficulty of on the fly 3D reconstruction and streaming of scenes from the real-word into the virtual one. However, a recent study by Lindlbauer and Wilson (2018) attempted to perform a live 3D reconstruction of the physical environment where a VR user was physically situated. The authors used eight Kinect cameras for a room-scale coverage to stream scenes from the real word.

Mixed Reality (MxR) blends the real and virtual environments in different forms and proportions. MxR applications are emerging in the VH domain following the recent advances in immersive reality technologies. For instance, Pollalis et al. (2018) presented a MxR application that utilizes Microsoft HoloLens to allow object-based learning through mid-air gestural interaction with virtual representations of museum artifacts. Similar to AV, MxR applications are not common in VH. There are a number of valid reasons as to why this is the case. First, the technological requirements of blending real and virtual elements to the extent that the blend appears as real as the real environment is extremely challenging. Second, MxR has been understood as a variation of AR or a fusion of AR and VR rather than a self-standing form of immersive reality (Piumsomboon et al., 2019). Third, AR and VR have been considered as the default immersive reality technologies in the domain (Haydar et al., 2011; Papagiannakis et al., 2018). As a result, VH has been adopting these technologies following their growing popularity rather than predetermining their relevance or comparing their potential against the intended VH application's requirements, which our paper attempts to achieve.

### Enabling Technologies of Immersive Reality

**Figure 1:**
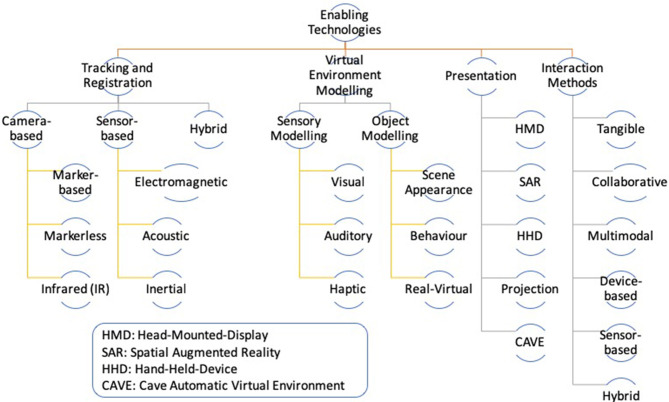
Enabling technologies of immersive reality, summarized from Bekele et al. (2018).

The immersive reality categories discussed above rely on and benefit from display technologies, tracking and registration mechanisms, interaction methods, and virtual environment modeling techniques (Billinghurst et al., 2015; Bekele et al., 2018; Kim et al., 2018). Interested readers can refer to these papers for detailed discussion on the enabling technologies. However, the sections below will briefly discuss these essential aspects of immersive reality. Figure 1 will also summarize the discussion.

#### Tracking and Registration

Tracking refers to the process of determining users' viewpoint position and orientation. Immersive reality systems require tracking to superimpose and display virtual information relative to users' or the camera's viewpoint position. In general, there are three categories of tracking techniques commonly used in immersive reality. Those are camera-based, sensor-based, and hybrid tracking methods.

- Camera-based tracking uses a digital camera, vision algorithms, and markers (markers can be in printed forms or infrared emitting devices). Camera-based tracking has two variations. The first one requires markers that need to be attached to a target and the vision algorithm determines the pose of the target that has a marker detected though the camera. The second variation of camera-based tracking relies on markerless and inside-out tracking mechanisms. A typical example is the environmental understanding and tracking cameras in Microsoft HoloLens.
- Sensor-based tracking uses different types of sensing devices, such as electromagnetic, acoustic, and inertial sensors installed at a base station and measurement points. Under this category tracking relies on measuring the intensity of signals and the time taken by the sensors to transmit and receive signals.
- Hybrid tracking is a combination of different tracking devices and techniques, such as GPS, Inertial Measurement Unit (IMU), motion sensors, and eye tracking.

#### Audio-Visual Presentation Technology

Presentation devices are the core of immersive reality. Based on the type of the virtual content, presentation devices are further classified into visual, auditory, and tactile presentation devices. This paper, however, discusses visual display devices, because most of existing visual display technologies are also capable of audio content presentation. There are five types of displays in this category: Head-Mounted-Display (HMD), Spatial Augmented Reality (SAR), Hand-Held-Devices (HHD), desktop screen and projection, and Cave Automatic Virtual Environment (CAVE).

- HMDs are highly immersive and commonly utilized across all immersive reality categories. Usually, HMDs made for AR and/or MxR are either video or optical see-through, whereas HMDs built for VR and/or AV experiences are blocked headsets since users' direct view to the physical environment is blocked.
- Spatial Augmented Reality (SAR) projects virtual information directly on the real environment through video-projectors. Two or more projectors are used for 3D effects.
- HHDs are portable displays such as smartphones and tablets. This group of displays have become a popular platform for mobile AR. These devices can also support VR if they are combined with additional VR kits such as Google Cardboard and RoboVR.
- Desktop screens and table-top projectors are common display systems for non-immersive VR and AR applications with a limited interactivity. These displays can provide 3D experiences with the addition of stereo glasses.
- The CAVE is a projection-based display technology that allows multiple co-located users to share fully immersive VR experiences. However, it is difficult to adjust the displayed content relative to all users at once, because tracking all users' pose and correcting the content's perspective to the tracked pose at the same time is challenging. Usually, a single user's pose is tracked to continuously correct the VR content's perspective relative to this user and the remaining users' experience is the same as the tracked user.

#### Interaction Methods

Interaction between users and virtual content is a crucial element of any immersive visualization environment. This is even more true for VH applications where cultural leaning is impacted by the interaction with virtual content. The common types of interaction methods are: tangible, collaborative, device-based, sensor-based, multimodal, and hybrid interaction methods.

- Tangible interfaces allow direct manipulation and interaction with virtual information through physical objects.
- Collaborative interfaces often use a combination of complementary interaction methods, sensors, and devices to enable a co-located and/or remote collaboration among users.
- Device-based interfaces use GUIs and conventional devices, such as mouse, gamepad, joystick, and wand to enable interaction and manipulation of virtual content.
- Sensor-based interaction interfaces use sensing devices to perceive users' interaction inputs. The common types of sensors include motion trackers, gaze trackers, and speech recognisers.
- Multimodal interfaces are a fusion of two and more sensors, devices, and interaction techniques that sense and understand humans' natural interaction modalities. This interface group allows gestural, gaze-based, and speech-based interaction with virtual content. Multimodal interfaces are closely related to sensor-based interfaces. However, the former combines multiple modes of interaction.
- Hybrid interfaces integrate a range of complementary interaction interfaces to devise a method that combines different characteristics from the above categories. For instance, a combination of collaborative, and multimodal interfaces.

#### Virtual Environment Modeling Methods

In general, the commonly used techniques of virtual environment modeling can be categorized into sensory modeling and object modeling methods. From a sensory modeling perspective, the methods are further classified into visual, auditory, and haptic sensorial modeling. From object modeling perspective, on the other hand, the methods are categorized into scene appearance, physics-based behavior, and real-virtual environment modeling (Zhao, 2009). Of these, scene appearance and real-virtual modeling methods are commonly used in VH applications, because the scene appearance modeling focuses on representing the geometric and spatial aspects of objects and the real-virtual modeling focuses on the interfusion of real and virtual scenery. When modeling virtual environments, there are three factors that need to be considered to determine the relevance of a method. Those are, complexity of objects in the real world, intended multimodality of interaction with the virtual environment, and the expected degree of model fidelity (Zhao, 2009). Furthermore, model data acquisition techniques such as photogrammetry and laser scanning are used to generate data for 3D reconstruction and simulation of cultural assets. Hence, an ideal approach to virtual environment modeling would be a combination of modeling methods and 3D data acquisition techniques.

## Comparing Immersive Realities and Interaction Interfaces

Virtual environments have the potential to serve as a platform that facilitates cultural learning (Ibrahim and Ali, 2018). Similarly, the importance of interaction methods for virtual environments to enable engagement and cultural learning has been emphasized (Tost and Economou, 2009; Champion et al., 2012; Caputo et al., 2016). Furthermore, it has been demonstrated that learning in virtual environments may not be achieved if the interaction method is not easy to operate or if the novelty of the interface overshadows the content (Economou and Pujol, 2007). Hence, balancing interaction, engagement, and content is very crucial for learning. More specifically, cultural learning relies on the contextual connection (relationship) between users and cultural context, and on some form of collaboration between users (Maye et al., 2017; Rahaman, 2018; McGookin et al., 2019; Šašinka et al., 2019).

Enhancing cultural learning in VH applications, therefore, requires the underlaying immersive reality and interaction method to enable a contextual relationship, collaboration, and engagement between users and the virtual environment (Champion, 2010; Jankowski and Hachet, 2013; Caputo et al., 2016; Rahim et al., 2017). This section will compare immersive reality technologies and the commonly used interaction methods against their potential to enable contextual relationship, collaboration, and engagement. The comparison attempts to establish a baseline to predetermine their relevance for disseminating cultural knowledge and enhancing cultural learning in VH applications.

The first factor, relationship, refers to establishing a contextual relationship between users, cultural context, and the immersive reality systems. Existing VH applications that adopt immersive reality technologies for cultural knowledge dissemination focus on users' interaction with the VH applications (Ridel et al., 2014; Schaper et al., 2017; tom Dieck and Jung, 2017; Caggianese et al., 2018). However, in order for VH applications to enhance cultural learning, establishing a contextual relationship between users, their physical surroundings (museums and heritage sites), and the virtual environment (cultural content) is as crucial as enabling intuitive interaction with the virtual environment. Hence, the relationship factor can be further categorized into three: relationship between user and reality (User-Reality relationship), relationship between user and virtuality (User-Virtuality relationship), and relationship between reality and virtuality (Reality-Virtuality relationship). An ideal immersive reality scenario will combine these subfactors into a User-Reality-Virtuality (URV) relationship (Bekele and Champion, 2019).

The second factor, collaboration, denotes the capability of a virtual environment to allow either a co-located or remote collaboration between a minimum of two users. Collaboration can be considered as both an aspect of VH experience and a form of interaction method. In both cases, the collaborative environment/method mimics or it reflects users' or visitors' experience as it would be at physical museums or heritage sites. Enabling collaboration requires more than a collaborative interaction with a virtual simulation/reconstruction of cultural heritage. It also requires the implemented VH application to influence users' experiential aspects as a result of their collective actions.

The third factor, engagement, is related to the ability of the virtual environment to enable engaging experiences as a result of the combination of immersivity and intuitive interaction with the cultural context in the virtual environment. To this end, VH applications rely on interaction methods, immersive headsets, and relevant cultural context. For instance, combining a tangible interaction method with highly immersive virtual environment and a relevant cultural context can be as engaging as a physical visit in museums and heritage sites (Katifori et al., 2019). Hence, VH applications that balance cultural context, interaction, and immersivity can lead to enhanced cultural learning.

In summary, whether cultural learning can be enhanced in VH applications depends on the capability of the different forms of immersive reality technology and interaction methods to enable contextual relationship with users, reality (cultural asset) and virtuality (virtual content), enable collaboration between users, and enable engagement with both the cultural context and virtual environments.

### Immersive Realities for Virtual Heritage Applications

In general, immersive reality technologies enable user-centered and personalized presentation of VH and make cultural heritage digitally accessible. The accessibility can be realized in a form of virtual reconstruction, simulation, or virtual museums. Such characteristic is viable, especially when physical access to artifacts is limited. In addition to increasing accessibility, immersive reality technologies can enhance cultural learning and enable visitors to have their own interpretation of cultural assets (Dow et al., 2005; Chrysanthi et al., 2012; Baldissini and Gaiani, 2014; Bustillo et al., 2015; Chang et al., 2015). In line with the potential and demonstrated capability of immersive reality to enhance learning in virtual environments, our paper attempted to compare current immersive reality technologies aiming at making suggestions as to which technologies can benefit VH applications. Hence, a detailed comparison of immersive reality technologies against the three factors (relationship, collaboration, and engagement) is attempted.

The comparison is performed by carefully assessing whether a given immersive reality technology or interaction method can enable the following:

- Engagement: does the technology or method enable engagement? What is the level of engagement supported?
- Co-located collaboration: does the technology or method support co-located collaboration?
- Remote collaboration: does the technology or method support remote collaboration?
- Relationship between users and virtuality: does the technology or method enable interaction and relationship between users and virtuality?
- Relationship between reality and virtuality: does the technology or method enable interaction and relationship between reality and virtuality?
- Relationship between users and reality: does the technology or method enable interaction and relationship between user and reality?

**Figure 2:**
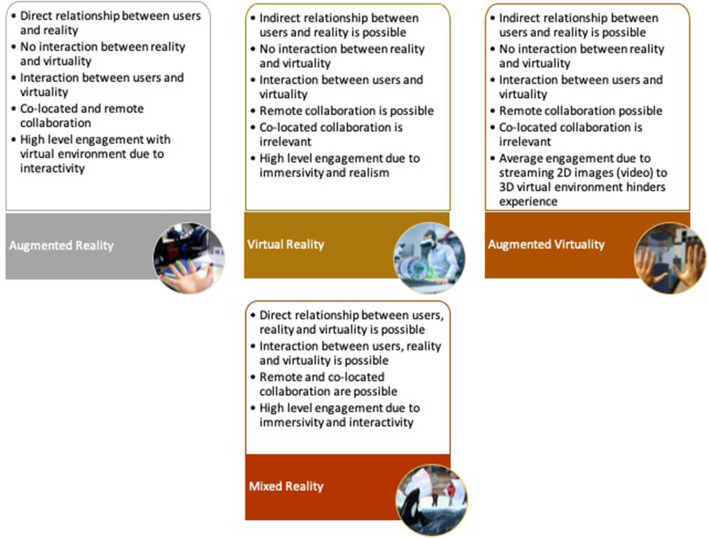
Identified capabilities of immersive reality technologies based on a comparison of their features against the three criteria (relationship, collaboration, and engagement). This figure is a summary of Table 3.

**Figure 3:**
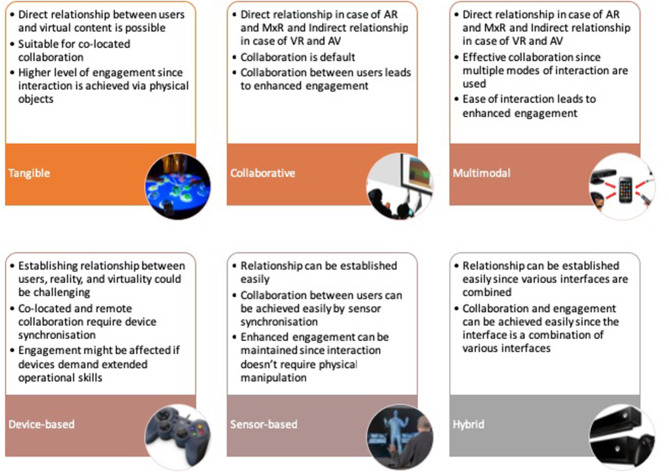
Identified capabilities of current interaction methods based on a comparison of their features against the three criteria (relationship, collaboration, and engagement). This figure is a summary of Table 4.

Taking the above questions into consideration, the assessments performed on the current immersive reality technologies and interaction methods are presented in Tables 3, 4, respectively. Furthermore, the assessments are summarized as presented in Figures 2, 3, Tables 1, 2 to make the details more presentable.

**Table 1:** Comparison of immersive reality technologies (summary of Table 3).

**Comparison factors**	**Immersive reality technologies**			
	**AR**	**VR**	**AV**	**MxR**
Engagement	High	High	Average	High
Co-located collaboration	Yes	No	No	Yes
Remote collaboration	Yes	Yes	Yes	Yes
Relationship (User-Virtuality)	Yes	Yes	Yes	Yes
Relationship (Reality-Virtuality)	No	No	No	Yes
Relationship (User-Reality)	Yes	No	No	Yes

**Table 2:** Comparison of interaction methods (summary of Table 4).

**Comparison factors**	**Interaction methods**					
	**Tangible**	**Collaborative**	**Multimodal**	**Device-based**	**Sensor-based**	**Hybrid**
Engagement	High	High	High	Average	High	High
Co-located collaboration	Yes	Yes	Yes	Yes	Yes	Yes
Remote collaboration	No	Yes	Yes	Yes	Yes	Yes
Relationship (User-Virtuality)	Yes	Yes	Yes	Yes	Yes	Yes
Relationship (Reality-Virtuality)	No	Yes	Yes	Yes	Yes	Yes
Relationship (User-Reality)	Yes	Yes	Yes	No	No	Yes

**Table 3:** A comparison of different forms of immersive reality technology against relationship, collaboration, and engagement: the comparison assists predetermining the relevance of a given form of immersive reality to enable cultural learning in virtual heritage applications.

**Immersive reality**	**Relationship**	**Collaboration**	**Engagement**
Augmented Reality (AR)	AR can establish a relationship between users and virtual content (cultural content)	Both remote and co-located collaborations can be implemented in AR	Engagement with the cultural content in virtual environments depends on the interaction interfaces' capability to enable intuitive interaction between users the virtual environment
	Interaction and relationship between users and their physical environment can be maintained since users view to the physical word isn't blocked. However, there is no direct relationship/interaction between the real world and virtual content, except virtual elements are superimposed over the real world		
	Interaction is always between users and virtuality (virtual content), and digital representations or simulations of cultural assets		Tangible and sensor-based interaction interfaces can enhance engagement since they pause relatively lower cognitive load
Virtual Reality (VR)	Interaction is always between users and virtual environments (cultural content)	Remote collaboration can be achieved by representing users as avatars in virtual environments	Virtual environments in VR are engaging due to their higher level of visual realism, immersivity, and presence
	There is no direct interaction/relationship between users and the real world because VR blocks users view to the real environment. However, indirect relationship can be established via virtual simulations and representations of cultural assets in the virtual environment	Collaborative VR isn't common in VH	Sensor-based and device-based interaction interfaces are employed commonly in current VR systems. However, device-based interfaces might hinder the level engagement because users are required to physically manipulate those devices, and this might cause a discontinuation of presence
Augmented Virtuality (AV)	Interaction is always between users and virtual environments (cultural content)	Remote collaboration can be achieved by representing users as avatars in the virtual environment or streaming a live video of users into the virtual environment. However, collaborative AV is extremely rare in any domain	Level of the virtual environment's realism and immersivity can directly determine the extent of engagement in AV
	Indirect relationship can be established between users and elements from the real world since live scenes are streamed from the real world to the virtual one		Usually, scenes streamed from the real world to the virtual environment aren't live 3D reconstructions. Hence, level of engagement could be hindered due to fusion of 2D and 3D images
	The relationship between elements from the real world and the virtual environment benefits the virtual environment		
Mixed Reality (MxR)	A symbiotic relationship can be maintained between the real and virtual environments by blending elements from both worlds	Co-located and remote collaboration can be implemented in MxR	Engagement is higher in MxR in contrast to the other forms of immersive reality since it can combine elements from both the real and virtual worlds. This means virtually reconstructed cultural content can be blended with cultural heritage elements at their natural location
	Unlike other forms of immersive reality, interaction and relationship can be established between users, reality, and virtual environments (cultural content)	MxR is an idea option for VH applications that require face-to-face collaboration at heritage sites and museums	Multimodal interaction interfaces that combine gestural, speech, and movement-based inputs can enhance user's engagement since the cognitive load of operating such interfaces is lower in contrast to other interfaces

**Table 4:** A comparison of different categories of interaction interfaces against relationship, collaboration, and engagement: the comparison assists predetermining the relevance of a given interaction interface to enable cultural learning in virtual heritage applications.

**Interaction methods**	**Relationship**	**Collaboration**	**Engagement**
Tangible	Tangible interaction interfaces use physical objects to enable interaction with virtual content. This provides suitable setting to establish a direct relationship between users and virtual reconstructions and representations of cultural elements	Co-located collaboration is better achieved with tangible interaction interfaces since users can interact with virtual content via collectively manipulating physical objects. However, this might add extra sophistication to the design and development process since the interface in such cases requires a capability to capture inputs from multiple users	Interacting with virtual content through physical objects enhances users' engagement in virtual environments
Collaborative	Collaborative interaction interfaces enable two or more users' collective actions to enable interaction with virtual environments. This characteristic makes collaborative interfaces a viable approach for establishing a relationship between users, virtuality (cultural content) and the physical environment	Collaborative interfaces are viable mainly for applications that require users to collaborate in order to interact with virtual content disseminated via immersive reality systems	Collaboration between users leads to enhanced engagement in virtual environments as the interface mimics how users interact with cultural heritage collections at heritage sites and museums
Multimodal	Multimodal interfaces enable interaction with virtual content via a combination of different modes of interaction. Gestural, movement, speech, touch, and gaze are the main modes of interaction in this interface	Collaboration between users is better achieved with multimodal interfaces since such interfaces are versatile and mimic how users would interact with their physical environment	Multimodal interfaces provide enhanced engagement due to the interface's ease of use resemblance to natural interaction
	Multimodal interfaces resemble how we interact with our physical environment. Hence, this group of interfaces enable users to establish a relationship with cultural context		
Device-based	Device-based interfaces enable interaction with virtual environments via haptic interfaces, and conventional devices, such as mouse, gamepad, joystick, and wand	Most devices in this category of interaction interfaces are designed for individual use. Hence, enabling collaboration across remote or co-located users requires synchronizing the devices, for instance, similar to collaborative video games	In general, device-based interfaces might affect engagement in virtual environments if the devices are demanding in terms the expertise required to operate them. This might interrupt users' presence in the virtual environment
	Enabling a contextual relationship between users, cultural context and virtual environments could be challenging since device-based methods require users to physically manipulate the devices		
Sensor-based	In general, sensor-based interfaces employ sensing devices to understand different modes of interaction, such as motion tracking and speech recognition. Usually, the interfaces sense users' intention to interact with virtual environments. Hence, these interfaces can effectively maintain an enhanced relationship between users, virtual environments and the cultural content embedded in the environment	Collaboration can be achieved easily by synchronizing multiple sensors. However, current sensor-based interfaces target individual users	Engagement in virtual environments could be higher since the interface doesn't require physical manipulation. This results in a reduced effort to operate the interface and a higher level of engagement
Hybrid	Hybrid interfaces integrate two or more types of interfaces discussed above. As a result, a continuous relationship between users, cultural assets and virtual environments can be maintained by exploiting the strength of each interface	Hybrid interfaces' potential to exploit favorable features from other interfaces put them at a viable position to provide collaborative virtual environments	Hybrid interfaces can achieve a higher level of engagement by integrating collaborative and multimodal interfaces

#### Mixed Reality (MxR)

Mixed Reality (MxR) is a unique form of immersive reality in a sense that it can provide, if exploited properly, a symbiotic platform where all the three criteria (relationship, collaboration, and engagement) can be balanced to benefit both the real and virtual environments. A contextual relationship between users, reality (cultural elements from the physical environment), and virtual content (3D reconstruction and simulation) can be maintained. This puts users at the center of the experience, affects their senses, and allows users to be part of any change and process in the real-virtual environment. This technology's potential to merge real and virtual elements enable the virtual environment to appear as real as the real. The real-virtual environment helps to enhance our understanding of both worlds, meaning the virtual elements enhance the real world and elements from the real world enhance the virtual one. From a VH perspective, this translates into merging 3D recontractions of lost tangible and intangible heritage elements with their currently remaining portions or natural locations and establishing a relationship between users and the merged environment.

In addition, MxR enables both co-located and remote collaboration. Remote collaboration can be implemented in all forms of immersive reality technology. However, a co-located collaboration is achieved only through AR and MxR, because this kind of collaboration requires users' local collective actions when interacting with the virtual environment. Even if both AR and MxR enable a co-located collaboration, MxR can add immersivity to the experience. Hence, VH applications that require some form of collaboration between users can benefit from a MxR technology.

Another feature that puts MxR ahead of AR, VR, and AV is engagement. This experiential aspect can be applied easily in MxR than the other forms of immersive reality, because MxR can combine elements from both the real and virtual worlds. This means virtually reconstructed cultural content can be blended with physical cultural heritage elements at their natural location. All in all, MxR is a viable form of immersive reality to create a VH experience that exhibits the three criteria (relationship, collaboration, and engagement) in order or enhance cultural learning.

It could be argued that AR can enable VH applications to exhibit the same properties as much as MxR does, because both conventionally attempt to enhance our understanding of the physical world by superimposing digital information over our view of the physical environment. However, these two forms are markedly different from experiential and technological perspectives. For instance, AR can't enable a symbiotic relationship between the physical and the virtual environments, it is always the physical environment the avails from the relationship. MxR, on the other hand, enables a symbiotic relationship and interaction between the real and virtual environments. As such, contextual relationship, collaboration, and engagement can be easily implemented in MxR.

#### Virtual Reality (VR)

Virtual Reality (VR) is highly immersive and transports users to a fully computer-generated world. From a VH perspective, such characteristic enables the reimagination an reconstruction of lost cultures in a highly immersive virtual environment. Interaction in VR is always between users and virtual environments (cultural content). There is no direct interaction/relationship between users and the real world, because VR blocks users' view to the physical environment. However, indirect relationship can be established via virtual simulations and representations of the physical world (or some elements from the physical world) in the virtual one.

The fact that users are blocked from the real-world view makes co-located collaboration less relevant to apply in VR. Even if it isn't commonly implemented in VH, remote collaboration can be achieved by representing users as avatars in virtual environments. Of all immersive reality segments, the virtual environments in VR are highly engaging due to their higher level of visual realism, immersivity, and presence. However, all the three criteria can't be balanced in VR—direct relationship between users and the physical environment can't be established, and co-located collaboration is irrelevant in VR since users are blocked from the real world. As such, VR's applicability to VH isn't as versatile as MxR. However, VH applications that don't require merging virtual elements and the physical environment and applications that attempt to reconstruct and simulate cultural heritage elements in a highly immersive virtual environment benefit from VR.

Similar to the close alignment of AR and MxR in terms of their objective, it could also be noticed that VR and AV share a similar goal of transporting users to a computer-generated virtual environment. However, VR and AV shouldn't be perceived as alternates for two main reasons. Firstly, the primary objective of virtual environments in VR is transporting users to a highly immersive and completely computer-generated world in which the user has no chance of establishing a direct relationship and interaction with the physical world. Hence, VR can achieve a higher sense of presence since the user isn't intermittently reminded of the physical environment. AV, on the other hand, streams live scenes from the physical world to the virtual one. This is problematic because it is technically challenging to perform a real-time 3D reconstruction and streaming elements from the real world to AV environments at the same time. Hence, AV applications end up streaming the physical world in 2D and this hinders users' presence and experience. Secondly, even if it is possible to stream 3D scenes from the physical environment, user's interaction and relationship is only with the virtual environment.

### Interaction Interfaces for Virtual Heritage Applications

The primary role of conventional interaction methods is to enable users to interact with computer systems. From a VH perspective, however, interaction interfaces play a huge role to create a contextual relationship between users and what the virtual environments represent. Hence, adopting interaction interfaces into VH applications needs predetermining whether a given method meets this expectation. However, it isn't common to come across to VH applications where interaction methods have been selected or customized based on their potential to establish a contextual relationship between users, cultural context and their potential to enable collaboration and engagement. Nevertheless, there are few exemplar cases of VH applications that have effectively used custom-made collaborative, multimodal and hybrid interfaces (Christou et al., 2006; Santos et al., 2010; Huang et al., 2016). In this regard, our paper attempts to compare different categories of interaction methods against the three criteria (relationship, collaboration, and engagement) that VH applications need to exhibit in order to enhance cultural learning. A detailed comparison is presented in Table 4 and summarized in Figure 3 and Table 2. Following the comparison, collaborative, multimodal, and a hybrid method that combines both were selected for further discussion based on their relevance for enhancing cultural learning in VH applications.

#### Collaborative Interaction Interface

Collaboration is a default feature in collaborative interaction methods. Such methods require an integration and synchronization of input devices, sensors, and audio-visual presentation technologies, such as gesture sensors, speech recognisers and HMDs (Piumsomboon et al., 2019). The ultimate goal of collaborative interaction is to enable a multiuser interaction with a shared virtual environment, meaning the interaction method has a technical and experiential aspects. For instance, two co-located users interacting with an identical virtual environment aren't necessarily interacting collaboratively unless the users' experience emanates from identical and a shared virtual environment. Hence, collaborative interaction, from an experiential perspective, requires users to interact with a shared, identical, and synchronized virtual environment. In addition, the users' collective or individual act of interaction needs to impact the virtual environment for all users. From a technical point of view, collaborative methods need: (1) devices and sensors that can acquire inputs from multiple sources, (2) visual, audio, or some form of cues to inform users when there is any act of interaction being performed by one of the collaborating users, and (3) synchronizing changes in the virtual environment.

Collaborative interaction interfaces can easily establish a contextual relationship between users and cultural content and can add a social dimension to the experience. In this regard, a study by Šašinka et al. (2019) indicates the importance of adding a social dimension to enhance learning in a collaborative and interactive visualization environment. Collaboration between users, therefore, leads to enhanced engagement in virtual environments as the interaction method mimics how users interact with cultural heritage collections and artifacts in museums and heritage sites.

#### Multimodal Interaction Interface

Multimodal interaction methods combine multiple modes of interaction, such as speech, gaze, gesture, touch, and movement. To this end, multimodal interfaces use a combination of sensors and devices to perceive humans' natural interaction modalities. Multimodality in immersive reality technologies can be perceived as a multisensorial experience and multimodal interaction. A multisensorial experience refers to users' visual, auditory, kinaesthetic, and tactile senses being affected by the virtual environment and interaction method. A multimodal interaction, on the other hand, explicitly refers to the use of multiple modes of interaction. However, a multisensorial experience is implicit in a multimodal interaction method.

Furthermore, multimodal interaction methods resemble how we interact with our physical environment. Hence, from a VH perspective, this group of interfaces enable users to establish a contextual relationship and collaboratively interact with the virtual environment. In addition, these interfaces enable VH applications to provide enhanced engagement with virtual environments and cultural context due to the method's ease of use and resemblance to natural interaction modalities.

### Hybrid (Collaborative Multimodal) Mixed Reality for Virtual Heritage

The main objective of collaborative and multimodal interaction methods is enabling collaboration between users and providing intuitive and natural interaction. Here, it is worth it differentiating collaborative interaction method and collaboration in virtual environment. The former explicitly refers to interaction methods/interfaces designed and implemented for collaborative interaction, meaning the interaction methods are designed to target more than one user at a time. Collaboration in virtual environments, on the other hand, refers to the experiential aspect of multiple remote or collocated users' interacting with a given virtual environment. The collaboration itself can be synchronized or asynchronized. The experiential aspect of collaboration in virtual environment is, therefore, implicit in collaborative interaction methods and interfaces. Recent advances in immersive reality technologies, such as the Microsoft HoloLens, are equipped with the necessary technology to enable the implementation of collaborative and multimodal interfaces in VH applications. However, collaborative and multimodal interfaces are still in experimental phases (Funk et al., 2017; Rahim et al., 2017). Furthermore, virtual environments that integrate customized interaction methods into the experience have been attempted (Damala et al., 2016; Signer and Curtin, 2017; Katifori et al., 2019) recently.

**Figure 4:**
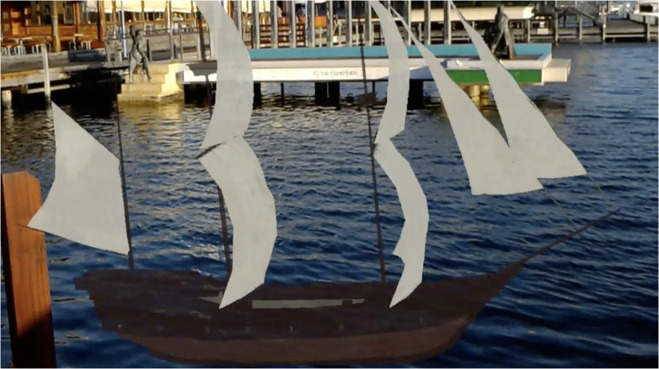
A mixed reality scenario showing a virtual ship merged with the physical environment at Fremantle, Western Australia.

Considering similar studies in the past and the comparison presented in Tables 3, 4, this paper proposes a specific integration of collaborative and multimodal interaction methods into MxR. This approach can enhance cultural learning at heritage sites and museums. The enhancement can be realized by exploiting the potential of MxR to merge digital content (3D models, audio, different multimedia) with the physical world (physical artifacts and heritage sites). For instance, Figure 4 shows a MxR scenario where a virtual ship is blended with the physical world (water environment). Such fusions allow for the dissemination/presentation of virtual reconstructions and simulations of heritage assets at their natural locations. As a result, users will be able to establish a contextual relationship with the real-virtual space.

**Figure 5:**
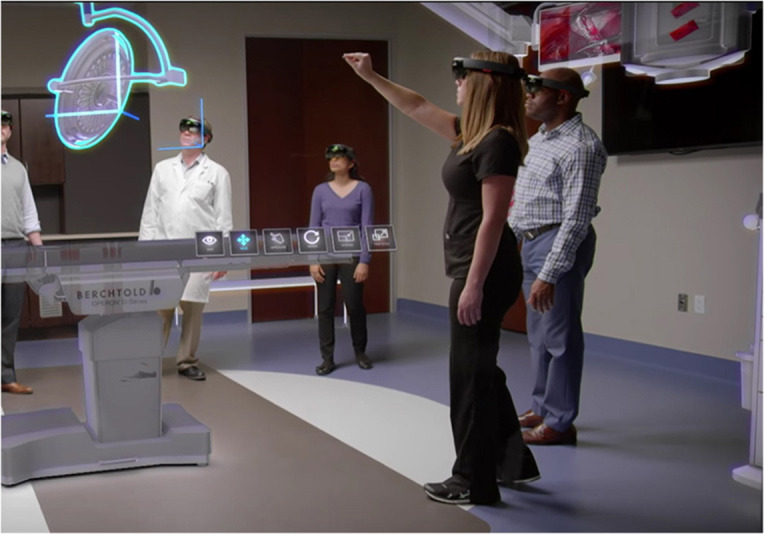
A mixed reality scenario showing five co-located users collaboratively interacting with a virtual environment (Image source, Microsoft).

Furthermore, adding a collaborative and multimodal interaction method to the MxR environment enables a face-to-face collaboration and distribution of interaction tasks among users. Distributing interaction tasks reduces the cognitive load on each members of a group. This leads to enhanced cultural learning since learning in virtual environments is directly impacted by users' effort to interact with the immersive system (Champion, 2006; Wang and Lindeman, 2015). Collaborative MxR reduces the impact since interaction is achieved with less effort from individuals as tasks are distributed among the group members. For instance, Figure 5 shows a collaborative MxR scenario where five co-located users interact with a shared virtual environment. In addition, the multimodal interaction enables enhanced interactivity and engagement since multiple modes of interaction, such as gaze, movement, speech and gesture, are used to interact with the collaborative MxR environment.

All in all, the proposed approach (Collaborative Multimodal Mixed Reality) can enhance cultural learning by: (1) establishing a contextual relationship between users, the virtual environment and the cultural context, (2) enabling collaboration between users, and (3) increasing the engagement with the virtual environment and the cultural context. To this end, the following technologies can be utilized to enable collaborative and multimodal interaction in MxR environments the primary attempt to enhance cultural learning in VH scenarios.

- Microsoft HoloLens is an HMD primarily designed and built for AR and MxR applications. The device has inbuilt environmental understanding cameras to track users and virtual objects' pose relative to physical objects from their immediate physical environment. In addition, the device has graphics-optimized processing unit.
- Microsoft has developed a development toolkit (Mixed Reality Toolkit) that can be integrated with Unity, which is a popular game engine supporting more than 25 platforms, to develop and deploy MxR application easily to HoloLens.
- Enabling collaboration and multimodality requires synchronization between at least two HMDs (HoloLens). This requires sharing pose, views and virtual objects' location and current state between collaborating users. To this end, cloud services, such as Microsoft Azure Spatial Anchor, Microsoft Azure Cosmos DB and Microsoft Azure Application Service can be used in combination to enable synchronization and sharing virtual objects' pose and current state.

A detailed system architecture, design and implementation of the Hybrid Mixed Reality system proposed above is being performed and the we are currently preparing an article that reports on the first phase of the implementation.

## Conclusion

In this paper, we have attempted to discuss different categories of immersive reality (AR, VR, AV, and MxR) and their enabling technologies from a VH perspective. We have also attempted to compare these immersive reality categories against their potential to establish a contextual relationship between users, reality, and virtuality and their capability to enable collaboration and engagement in virtual environments. In addition, we have attempted a similar comparison on different interaction methods (tangible, collaborative, multimodal, sensor-based, device-based, hybrid interfaces) in order to identify the best approach from an experiential and technological requirements perspective. Following the comparison, we have identified MxR and VR as potential categories of immersive reality. From the interaction point of view, collaborative and multimodal interaction methods were identified as viable approaches. Finally, we have proposed a specific combination of MxR and a hybrid interaction method comprising collaborative and multimodal features in order to enhance cultural learning at heritage sites and museums. This specific combination can be a practical approach for VH applications to establish a contextual relationship between users and cultural context and implement collaborative experience to add social dimension to the experience. Moreover, it can improve users' engagement with the virtual environment. As an extension to this paper, we plan to present a detailed design and implementation of the proposed approach.

## Author Contributions

Conceptualization, investigation, resources, and writing original draft by MB. Review and editing by MB and EC. Supervision by EC.

### Conflict of Interest

The authors declare that the research was conducted in the absence of any commercial or financial relationships that could be construed as a potential conflict of interest.
